# Study on the Stability of the Electrical Connection of High-Temperature Pressure Sensor Based on the Piezoresistive Effect of P-Type SiC

**DOI:** 10.3390/mi12020216

**Published:** 2021-02-21

**Authors:** Yongwei Li, Ting Liang, Cheng Lei, Qiang Li, Zhiqiang Li, Abdul Ghaffar, Jijun Xiong

**Affiliations:** 1 Science and Technology on Electronic Test & Measurement Laboratory, North University of China, Taiyuan 030051, China; liyongwei27@163.com (Y.L.); leicheng@nuc.edu.cn (C.L.); snjk08@163.com (Q.L.); lizhiqiangnuc@163.com (Z.L.); 92ghaffar@gmail.com (A.G.); 2 Department of Automation, Taiyuan Institute of Technology, Taiyuan 030051, China

**Keywords:** silicon carbide, pressure sensor, piezoresistive effect, mesa varistor, ohmic contact

## Abstract

In this study, a preparation method for the high-temperature pressure sensor based on the piezoresistive effect of p-type SiC is presented. The varistor with a positive trapezoidal shape was designed and etched innovatively to improve the contact stability between the metal and SiC varistor. Additionally, the excellent ohmic contact was formed by annealing at 950 °C between Ni/Al/Ni/Au and p-type SiC with a doping concentration of 10^18^cm^−3^. The aging sensor was tested for varistors in the air of 25 °C–600 °C. The resistance value of the varistors initially decreased and then increased with the increase of temperature and reached the minimum at ~450 °C. It could be calculated that the varistors at ~100 °C exhibited the maximum temperature coefficient of resistance (TCR) of ~−0.35%/°C. The above results indicated that the sensor had a stable electrical connection in the air environment of ≤600 °C. Finally, the encapsulated sensor was subjected to pressure/depressure tests at room temperature. The test results revealed that the sensor output sensitivity was approximately 1.09 mV/V/bar, which is better than other SiC pressure sensors. This study has a great significance for the test of mechanical parameters under the extreme environment of 600 °C.

## 1. Introduction

With the booming of control science and intelligent monitoring technology, the pressure sensor technology in extreme environments such as engines and oil drilling has received extensive attention from scholars [1,2]. Compared with capacitive, optical fiber, surface acoustic wave, and other types of sensors, piezoresistive pressure sensors have the advantages of the easiness of design configuration, small size, simple processing technology, and the wider linearity range [3,4,5]. Owing to the excellent piezoresistive effect of silicon (Si) and mature Si-based micro-electromechanical system (MEMS) processing technology, pressure sensors based on the Si-piezoresistive effect are currently the most commonly used. However, in a high-temperature environment above 500 °C, Si materials will undergo plastic deformation after pressure and are easily corroded or oxidized, limiting the application of Si pressure sensors in extreme environments [6,7,8].

As the third-generation, wide-band gap semiconductor, silicon carbide (SiC) has the advantages of the wide-band gap, high thermal conductivity, high mechanical strength, strong radiation resistance, and so on. It has been widely used to prepare sensors and power electronic devices working in extreme environments such as high temperature, high frequency, and high pressure [9,10,11]. What is noteworthy is that hexagonal SiC as bulk SiC, such as 6H- and 4H-, is considered the most promising semiconductor materials for the preparation of all SiC sensors working in high-temperature environment semiconductors [12,13]. The piezoresistive properties of SiC have attracted more and more attention from scholars, along with the development of SiC crystal growth technology, homogeneous epitaxy technology, and doping technology [14,15]. However, compared with the piezoresistive properties of Si, the strain coefficient of SiC is about 5 times smaller and the temperature coefficient of gauge factor (TCGF) is larger, which leads to the generally lower sensitivity of pressure sensors based on the piezoresistive effect of silicon carbide. Robert S. Okojie et al. verified the significant piezoresistive effect of 6H-SiC and prepared the high-temperature pressure sensor that could work at 600 °C in 2007. Nevertheless, due to the insufficient strain coefficient and excessive temperature coefficient of gauge factor (TCGF), the sensor’s output sensitivity deteriorated seriously in the environment of more than 300 °C [16,17,18]. In order to promote the application of SiC piezoresistive pressure sensors in high-temperature environments, some teams have turned their research interests to 4H-SiC.

In 2012, T. Akiyama et al. reported for the first time that the strain coefficient of n-type 4H-SiC was approximately 20.8 [19]. In 2015, Robert S. Okojie prepared a pressure sensor based on the n-type 4H-SiC piezoresistive effect that can work at 800 °C. The sensor’s output sensitivity gradually decreased when it was less 400 °C, increased when it was more significant than 400 °C, and returned to where the values measured at 800 °C were nearly equal to or higher than the room temperature values. However, the sensor’s dynamic performance was poor, which may be caused by insufficient strain coefficient [20]. After that, Tuan-Khoa Nguyen et al. from Griffith University began to investigate the characterization of the p-type 4H-SiC piezoresistive effect. The team experimentally confirmed for the first time that the strain coefficient of p-type 4H-SiC was about 31.5, which was 1.5 times of the strain coefficient of n-type 4H-SiC [21]. Meanwhile, Li et al. from the North University of China also obtained similar results [22]. It is worth noting that the SiC epitaxial layer’s square resistance in the references [21,22] was about 22.9 kΩ/☐ and 5.6 kΩ/☐, respectively, which was too large for the preparation of sensors. Analysis showed the large square resistance was caused by the poor ohmic contact effect between metal and SiC, consistent with the literature’s viewpoint [23]. To the best of our knowledge, there are few articles on the design and fabrication of sensors based on the p-type 4H-SiC piezoresistive effect.

In this paper, a pressure sensor based on the p-type 4H-SiC piezoresistive effect was designed and prepared. In order to improve the stability of the electrical connection between the metal leads and varistors, a positive trapezoidal mesa resistor was innovatively proposed. Furthermore, Ni/Al/Ni/Au and p-type silicon carbide (SiC) with an aluminum doping concentration of 8.1 × 10^18^ cm^−3^ formed good ohmic contact after annealing at 950 °C under vacuum for two minutes. The square resistance of the SiC epitaxial layer was about 448.5 Ω and the contact resistivity was about 10^−4^ Ω·cm^2^, measured using a rectangular transmission line model. The sensor chip was prepared according to the designed process flow, and varistors of the aging sensor were tested with the temperature. In the temperature range of 25 °C–600 °C, the varistors first decreased and then increased with the increase of temperature. At about 100 °C, the temperature coefficient of resistance (TCR) reached a maximum of –0.35 %/°C, which proved that there was a stable electrical connection between metal and SiC mesa resistors. Finally, a pressure/depressure test was carried out on the encapsulated sensor at room temperature. The sensor output sensitivity was ~1.09 mV/V/bar, which was better than most of the SiC pressure sensors that have been reported. This research laid a technical foundation for the development of pressure sensors based on the p-type SiC piezoresistive effect.

## 2. Materials and Methods

### 2.1. Sensor Design

**Figure 1:**
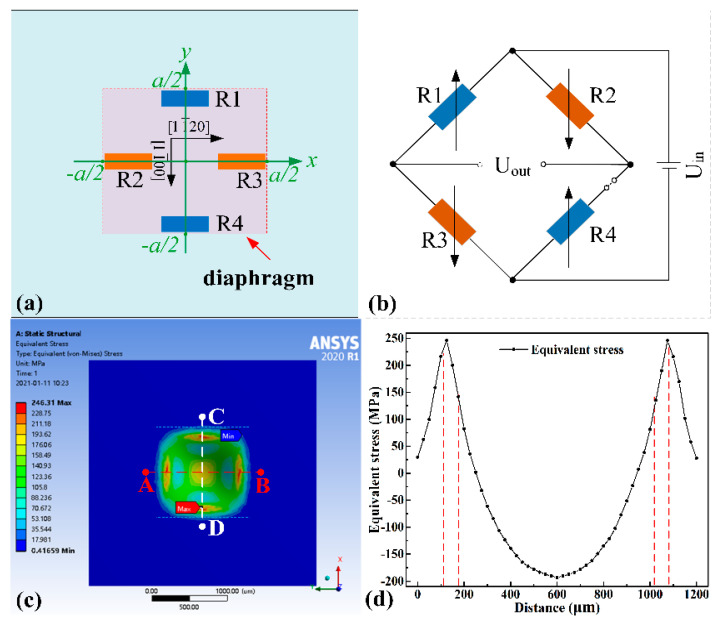
(**a**) Schematic diagram of sensor structure and varistor layout position. The diaphragm size is 1000 μm × 1000 μm × 45 μm and the sensor chip size is 3300 μm × 3300 μm × 346 μm; (**b**) connection method of the varistors; (**c**) the finite element simulation results of SiC pressure-sensitive structure when the pressure of 1 MPa is applied; (**d**) the stress distribution from path A to B, which is symmetric to path C to D.

The pressure sensor based on the piezoresistive effect integrates four varistors into a pressure-sensitive diaphragm and connects them to be a Wheatstone bridge. When the environmental pressure changes, the varistors on the diaphragm change in proportion to the pressure and the pressure signal is detected by the output of the bridge circuit. The standard pressure-sensitive diaphragm includes square diaphragms, circular diaphragms, and island diaphragms with masses’ block. With the difficulty of SiC MEMS processing, island diaphragms are generally not considered as pressure-sensitive diaphragms. According to the theory of elasticity, the diaphragm’s maximum stress under the same size condition is 1.64 times larger than that of the round diaphragm [24,25]. Therefore, in this paper, the pressure sensor adopted the square pressure-sensitive diaphragm of 1000 μm × 1000 μm. The plane structure model of the square diaphragm is shown in Figure 1(a), which was supported on four sides. According to the thin plate theory [24], the deflection distribution on the square diaphragm under pressure can be expressed as Equation (1).

(1) $$\omega (x,y)=0.0213\frac{16p}{a^{4}D}{\left(\frac{a^{2}}{4}-x^{2}\right)}^{2}{\left(\frac{a^{2}}{4}-y^{2}\right)}^{2}$$

where *ω*(*x*,*y*) is the deflection of any point on the diaphragm (*x* = (−*a*/2, *a*/2), *y* = (−*a*/2, *a*/2)), *p* is the applied pressure, and *a* is the edge length of the square diaphragm. D denotes the bending strength of the diaphragm, which can be expressed as Equation (2).

(2) $$D=Eh^{3}/12(1-\nu^{2})$$

where *E* is the elastic modulus of the SiC, *h* is the thickness of the sensitive diaphragm, and *v* is Poisson’s ratio of the SiC. According to Equation (1), it can be deduced that the maximum deflection occurs at the center of the diaphragm (*x* = 0, *y* = 0).

The equivalent stress distribution on the square diaphragm under pressure can be expressed as Equation (3).

(3) $$\sigma (x,y)=0.5112\frac{16p(1-\nu )}{a^{4}h^{2}}[(3x^{2}-\frac{a^{2}}{4}){\left(y^{2}-\frac{a^{2}}{4}\right)}^{2}-(3y^{2}-\frac{a^{2}}{4}){\left(x^{2}-\frac{a^{2}}{4}\right)}^{2}]$$

where σ(*x*,*y*) is the equivalent stress of any point on the diaphragm. According to Equation (2), it can be deduced that the maximum equivalent stress occurs at the middle area of the edge of the diaphragm such as (*x* = *a*/2, *y* = 0). The following is to design the diaphragm’s thickness with a working range of 1 MPa, which needs to consider the linear principle and the anti-overload principle. The so-called linear principle means that the maximum deflection of the sensitive diaphragm must be less than 1/5 of the diaphragm’s thickness when the sensor is subjected to full-scale pressure to ensure that the diaphragm works in the linear region. The relationship between the maximum deflection of sensitive diaphragm, working range, and diaphragm thickness can be expressed as Equation (4).

(4) $$\omega_{\max}=\frac{0.0213\times 12\times (1-\nu^{2})p_{\max}a^{4}}{16\cdot Eh^{3}}<\frac{h}{5}$$

where *ω*_max_ is the maximum deflection of the diaphragm after being pressured and *p*_max_ is the maximum working pressure of the sensor. The so-called anti-overload principle means that when the sensor is subjected to full-scale pressure, the maximum equivalent stress of the sensitive diaphragm must be less than 1/5 of the yield stress of the SiC to prevent irreversible deformation of the sensor due to excessive pressure. The relationship between the maximum stress of the sensitive diaphragm and the working range and diaphragm size can be expressed as Equation (5).

(5) $$\sigma_{\max}=\frac{1.0224\times (1-\nu )p_{\max}a^{2}}{4\times h^{2}}\leq \frac{\sigma_{y}}{5}$$

where σ_max_ is the maximum equivalent stress on the surface of the pressure-sensitive diaphragm and σ_y_ is the yield stress of SiC. Based on the above calculation results and process feasibility, the size of the pressure-sensitive diaphragm was designed to 1000 μm × 1000 μm × 45 μm and the sensor chip size was 3300 μm × 3300 μm × 346 μm, as illustrated in Figure 1a.

Four varistors should be arranged in the stress concentration area of the sensitive diaphragm as far as possible to improve the sensor’s output sensitivity. The sensor model was established via ANSYS (Pittsburgh, PA, USA) and the pressure of 1MPa was uniformly applied on the surface of the diaphragm. Based on the finite element simulation results, as shown in Figure 1c, the stress concentration area on the diaphragm’s surface was distributed in the middle area of the edge of the diaphragm, which was consistent with theoretical calculations. Hence, the four SiC varistors with a size of 80 μm × 20 μm, R1, R2, R3, and R4, respectively, were arranged on the edge of the sensitive diaphragm, as shown in Figure 1b. What calls for special attention is that R1 and R4 were arranged perpendicular to the edge of the diaphragm, while R2 and R3 were arranged parallel to the edge. The four resistors were connected to form a semi-open-loop Wheatstone Bridge, as shown in Figure 1b. It was to facilitate the measurement of the resistance value of each resistor. When the uniform pressure was applied to the diaphragm’s surface, the resistance value of R1 and R4 increased while the piezoresistance of R2 and R3 decreased, resulting in the output of the bridge changing in proportion to the pressure. Ideally, since the four varistors sizes were the same, the bridge was balanced and the output voltage was zero in the absence of pressure. When pressure was applied to the surface of the diaphragm, the resistance value of the varistors changed. Assuming that the four resistors’ changes were uniform, the output of the bridge can be expressed as Equation (6).

(6) $$U_{out}=\frac{\Delta R}{R}U_{in}$$

where ΔR/R is the rate of change of single varistor and *U_in_* is the bridge’s input. It can be calculated so that the output sensitivity of the sensor is shown in formula (7).

(7) $$S=\frac{1}{P}\frac{\Delta R}{R}=\frac{1}{P}\frac{\Delta \rho }{\rho }=\frac{1}{P}\cdot \frac{GF}{E}\cdot \sigma_{a}$$

where *P* is the pressure applied on the surface of the diaphragm, Δ*ρ*/*ρ* is the rate of change of resistivity, *GF* is the strain coefficient of the varistor, and σ_a_ is the average stress of the varistor. When *GF* takes 30, according to reference [21], the output sensitivity of the sensor can be calculated theoretically as 1.3 *mV/V/bar.*

### 2.2. Sensor Preparation

In this experiment, n-type 4H-SiC wafer with a doping concentration of 10^14^ cm^−3^ was used as the substrate, which was purchased from China Tianke Heda Co., Ltd. The thickness of the SiC substrate was 342 μm and the diameter was 4 inches. The p-type epitaxial layer with a doping concentration of 10^19^ cm^−3^ and a thickness of 2 μm was homogeneously grown on the silicon surface of the SiC substrate to prepare the varistors. There was an n-type buffer layer with a doping concentration of 10^18^ cm^−3^ and a thickness of 2 μm between the p-type epitaxial layer and the substrate, which was to form a PN junction between the varistors and the substrate and prevent the current flow of the varistors from leakage to the substrate. The SiC homogeneous epitaxy process was realized via the chemical vapor deposition process in Dongguan Tianyu Semiconductor Co., Ltd.

**Figure 2:**
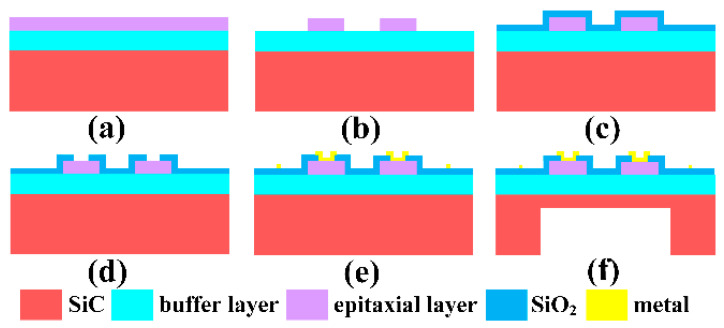
Process flow of sensor. (**a**) Structure diagram of SiC epitaxial wafer. (**b**) Varistors were shallowly etched using reactive ion etching machine (RIE). (**c**) The SiO_2_ was grown by thermal oxidation as an electrical isolating layer. (**d**) Ohmic contact holes were opened by wet corrosion of SiO_2_. (**e**) Metal electrodes were formed by metal deposition and metal stripping. (**f**) The pressure reference cavity of the sensor was deeply etched using inductively coupled plasma (ICP) machine.

The pressure sensor based on the piezoresistive effect of p-type 4H-SiC was manufactured using the standard MEMS process, as shown in Figure 2, including the crucial processes such as varistors’ etching, thermal oxidation, metal deposition, deep etching of pressure reference cavity, etc. Firstly, the SiC wafer with the p-type 4H-SiC epitaxial layer was cleaned using a standard root cause analysis (RCA) process. In order to remove impurities and suspended chemical bonds on the surface of the wafer, it was thermally oxidized at 1100 °C for four hours in the thermal oxidation furnace (Qingdao Huaqi Technology Co., Ltd., Qingdao, China) and then the SiO_2_ was corroded away in the buffered oxide etch (BOE) solution. Secondly, adopting AZ4620 photoresist as a mask, reactive ion etching machine (RIE) was used to etch the positive trapezoidal mesa varistors. The etching height of the varistors was about 2 μm. In order to effectively control the height of varistors, the SiC etching rate should not be too fast. The SiC etching rate corresponding to the RIE etching parameters shown in Table 1 was about 69 nm/min.

**Figure 3:**
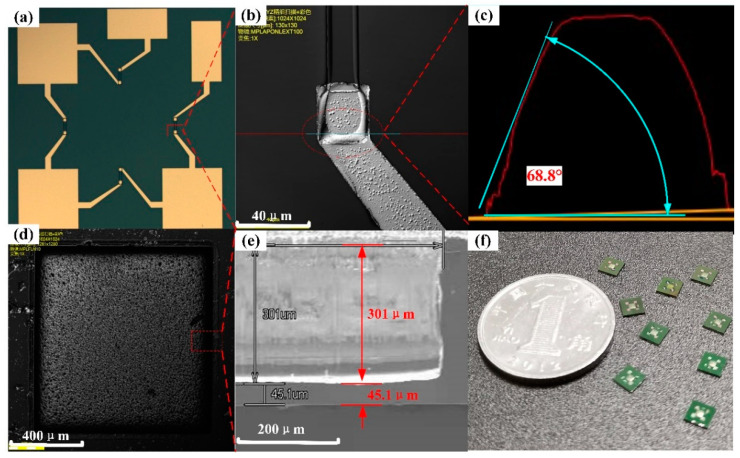
Diagram of sensor structure. (**a**) Front view of a single sensor chip. (**b**) The positive trapezoidal mesa resistor with metal lead. (**c**) Sidewall perpendicularity of mesa resistor. (**d**) Sensor pressure reference chamber image. (**e**) SEM image of the sidewall of pressure reference chamber. (**f**) Sensor chip sample.

What is worth mentioning was that the height of the varistors should be slightly larger than the thickness of the epitaxial layer, which is to ensure absolute electrical separation between each varistor. Therefore, it was necessary to test the p-type epitaxial layer’s thickness through secondary ion mass spectrometry (SIMS) before etching the mesa varistors. Thirdly, in order to ensure insulation between the metal lead and the SiC substrate, thermal oxidation was again used to grow the SiO2 insulation layer on the the wafer’s surface. Fourthly, adopting the AZ6130 photoresist as the mask, the oxide layer at both ends of the varistors was corroded away in the BOE solution to open the metal/SiC ohmic contact hole. Fifthly, the metal film of 20nm Ni/100 nm Al/10 nm Ni/200 nm Au was deposited by the magnetron sputtering equipment (Denton Vacuum Equipment Co., Ltd., Denton, TX, USA) after the lithography process patterned the metal electrode leads. The metal electrode was prepared by stripping the excess metal film in an acetone solution. It is worth noting that the use of oxygen plasma to etch the wafer’s surface before sputtering the metal not only increased the adhesion between the metal and SiC, but also contributed to the formation of the ohmic contact. Sixthly, the nickel mask of ~15 μm was prepared by electroplating process on the back of the wafer. Then the wafer was cut into 2 cm × 2 cm pieces, by DISCO DAD322 (an Automatic Dicing saw) (Disco Corporation, Tokyo, Japan), which were used to etch the pressure reference cavity by inductively coupled plasma (ICP) GDE C200 (North Microelectronics Company, Beijing, China). The parameters of the inductively coupled plasma (ICP) etching SiC are shown in Table 2 and the corresponding etching rate was ~1.4 μm/min. The pressure reference cavity was prepared by ICP etching for about 215 min, as illustrated in Figure 3d. As can be seen from the scanning electron microscope (SEM) image of the chamber’s sidewall shown in Figure 3e, the depth of the pressure chamber was about 301 μm, the thickness of the pressure diaphragm was about 45.1 μm, and the verticality of the sidewall was satisfactory.

According to the above technological process, the pressure sensor chip was prepared and the wafer was cut into individual chip using DISCO DAD322, as shown in Figure 3. Then, the annealing experiment was performed by using a rapid thermal processing furnace (Beijing East Star Institute of Applied Physics, Beijing, China) to explore the annealing parameters for forming the good ohmic contact between metal and p-type SiC. After that, the resistance values of the varistors were tested at 25 °C to 600 °C to verify the stability of the ohmic contact between the metal and SiC, as well as the reliability of the electrical connection between metal leads and the mesa varistors in the high-temperature environment. Finally, the wire bonding process was used for packaging the pressure sensor chip and the base, and the performance of the sensor was tested at room temperature.

## 3. Results and Discussion

**Figure 4:**
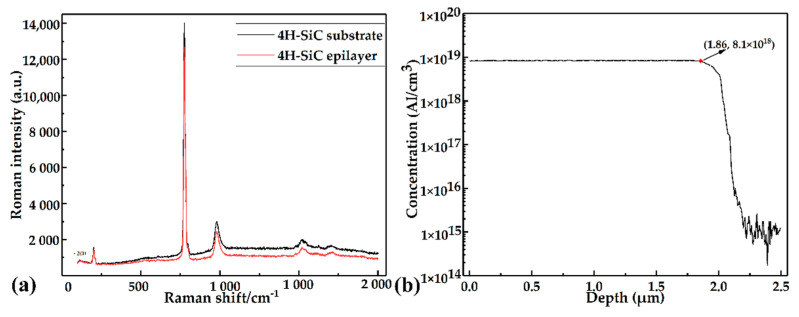
Characterization test of SiC epitaxial layer. (**a**) Raman spectra of epitaxial layer and substrate. (**b**) Secondary ion mass spectrometry (SIMS) pattern of the aluminum element in the epitaxial layer.

The p-type SiC epitaxial layer was patterned and then etched to prepare varistors, whose height was determined by the epitaxial layer’s actual thickness. More importantly, the quality of the epitaxial layer played a decisive role in the sensor’s performance. The Invial laser Raman spectrum analyzer was employed to scan the SiC substrate and the epitaxial layer. The crystal quality of the epitaxial layer was judged by comparing the spectral results. An Ar+ atomic laser with a power of 5 mW and a wavelength of 532 nm was used as the test system’s light source. After excitation, the laser was incident perpendicularly to the sample’s surface, and then the backscattered light was collected and analyzed via computer. The measured scattering spectra of the 4H-SiC substrate and epitaxial layer are shown in Figure 4a. It was observed that the substrate’s characteristic peaks were consistent with the epitaxial layer, confirming that the epitaxial layer belonged to the 4H-SiC crystal phase and had good quality. After that, the doping concentration and thickness of the epitaxial layer were tested using SIMS. The SIMS pattern is exhibited in Figure 4b. It was found that the concentration of doped aluminum in the epitaxial layer was ~8.1 × 10^18^/cm^3^ and the thickness of the epitaxial layer was ~1.86 μm. Therefore, the height of the mesa varistors was designed to be 2.0 μm. The positive trapezoidal varistors were obtained by RIE etching for 29 min, as shown in Figure 3b, and the sidewall perpendicularity of the mesa resistor was about 68.8°, as shown in Figure 3c, which was induced by controlling the topography of the photoresist mask. The angled mesa resistor increased the contact area between the metal lead and the SiC, improving the connection stability.

**Figure 5:**
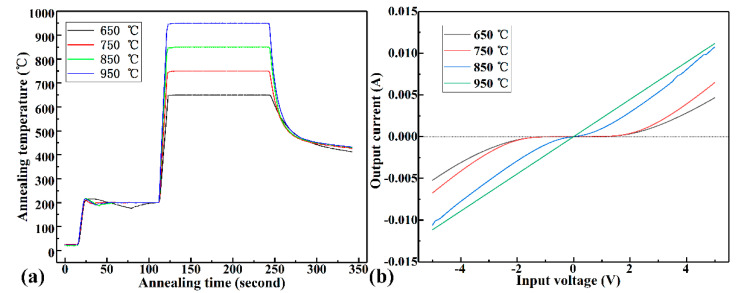
Ohmic contact test of SiC. (**a**) Annealing time–temperature curve. (**b**) The relationship between voltage and current at both ends of the resistor under different annealing conditions.

Generally, Ti/Al metal system is used to form an ohmic contact with p-type SiC, whereas it cannot be used in harsh environments because aluminum is easily oxidized and has a low melting point. Therefore, gold was deposited on the surface of Al as a protective layer. We employed 20 nm Ni/100 nm Al as the ohmic contact layer, 20 nm Ni as the isolation layer between metals, and Au as the electrode layer, which not only avoided oxidation of the metal electrode, but also facilitated wire bonding with external circuits. The metal electrode of the sensor is shown in Figure 3a. In order to form a good ohmic contact between the metal and SiC varistor, the sensor chip was annealed under different conditions. In this experiment, the sensor samples were quickly annealed in a vacuum environment for 2 min at 650 °C, 750 °C, 850 °C, and 950 °C. The curve of annealing temperature with time and the current–voltage characteristic curve at both ends of the varistor is shown in Figure 5, which was mesured by semiconductor parameter analyzer (Keithley, Cleveland, OH, USA). As can be seen in Figure 5b, when the annealing temperature was 950 °C, an excellent ohmic contact was formed. Using the rectangular transmission line model test, the square resistance and the specific contact resistivity of the SiC epitaxial layer were 448.5 Ω and 10^−4^ Ω·cm^2^, respectively.

**Figure 6:**
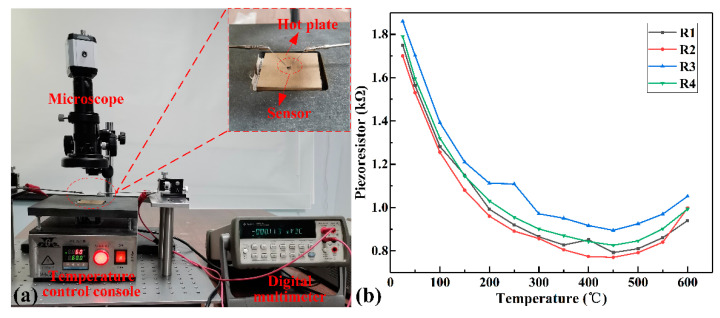
(**a**) Temperature control probe platform; (**b**) The variation curve of the varistors’ value with temperature.

In order to verify the stability of the metal/SiC ohmic contact and the reliability of the electrical connection between the metal lead and the mesa varistor in the high-temperature environment, the varistors of the sensor were measured within the temperature range of 25 °C to 600 °C. The test equipment mounted by us consisted of a temperature console, two probe consoles, a microscope, and a digital multimeter, as shown in Figure 6a. It is worth noting that the sensor samples were aged in a nitrogen atmosphere of 300 °C for 20 h before the resistances were tested. The resistance values of four varistors measured at room temperature were R1 = 1.75 kΩ, R2 = 1.70 kΩ, R3 = 1.86 kΩ, and R4 = 1.79 kΩ, respectively. Then, in the temperature range of 50 °C to 600 °C, the varistors’ change was tested by increasing/decreasing the temperature in steps of 50 °C. We read the multimeter data in the heating test experiment after holding each temperature point for 10 min. Furthermore, the lowering temperature test was carried out under natural cooling conditions. After averaging the two test results, the resistance value curve with temperature was plotted, as shown in Figure 6b. It can be seen from the test results that the stable transmission of electrical signals was achieved at temperatures from 25 °C to 600 °C, verifying the good connection between the metal lead and the mesa resistance. Additionally, the varistors’ resistance value obtained by increasing/decreasing the temperature tests was consistent, which implies a stable ohmic contact between the metal and the p-type 4H-SiC. Additionally, the TCR is a measure of the resistance changes with temperature, which is a pivotal parameter to determine the sensor’s ability to be used in high-temperature or low-temperature environments [26]. TCR can be expressed as Equation (8).

(8) $$TCR=\frac{1}{R_{0}}\frac{R_{t}-R_{0}}{T-T_{0}}$$

where *R*_0_ (Ω) is the resistance value at room temperature environment, *R*_t_ (Ω) is the resistance value at the operation temperature, *T* (°C) is the operating temperature, and *T*_0_ (°C) is the room temperature. According to the test results, the average TCR of the four p-type 4H-SiC varistors with the doping concentration of 8.1 × 10^18^ cm^−3^ in the range of 25 °C–600 °C can be calculated as shown in Table 3.

In combination with Figure 6b, it was found that the resistance decreased with the temperature increasing at less than 400 °C, indicating that the resistance was primarily determined by the degree of ionization of impurities below 400 °C. When the temperature exceeded 500 °C, the resistance increased with the increased temperature, indicating that lattice scattering played a significant role. Moreover, it was estimated that the impurities were entirely ionized between 400 °C and 500 °C.

**Figure 7:**
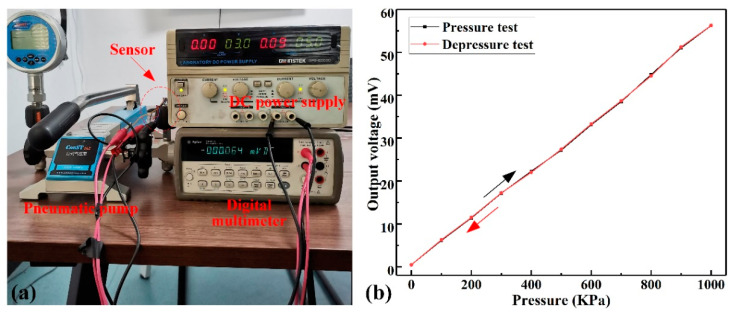
Performance test of the sensor at room temperature. (**a**) Pressure measuring system at room temperature. (**b**) The curve of output voltage as a function of pressure.

Next, the air tightness of the encapsulated sensor was tested by helium mass spectrometry. The sample was placed in a helium pressure chamber and kept under 600 KPa pressure for 2 h. The helium leakage rate was tested in the helium mass spectrometer’s sealed chamber to be 1.9 × 10^−9^ Pa·m^3^/s, meeting the requirements of most pressure sensors. The four varistors of the sensor were connected to a four-arm Wheatstone bridge and the 5-V input voltage was supplied using a direct-current (DC) power supply (Gwinstek, Taiwan). By increasing/decreasing the pressure in a step of 100 KPa within the pressure range of 0–1 MPa, the sensor’s output voltage was tested with a high-precision multimeter (Agilent Technologies Inc, Palo Alto, CA, USA). When the applied pressure reached 1 MPa, the backstroke test started to execute. The test equipment and the test results are inllustrated in Figure 7. Figure 7b indicates that the sensor’s output sensitivity was approximately 1.09 mV/V/bar at room temperature, which is better than the reported SiC pressure sensor [27,28,29,30]. The above experimental results confirmed that the pressure sensor based on the piezoresistive effect of p-type SiC has the potential to be applied in extreme harsh environments.

## 4. Conclusions

In this work, a high-temperature pressure sensor based on the piezoresistive effect of p-type SiC was designed and fabricated. In order to improve the stability of the electrical connection of sensor chips, the positive trapezoidal varistor was proposed. Using Ni/Al/Ni/Au as the metal system, the excellent ohmic contact was formed with p-type SiC annealed at 950 °C for 2 min in a vacuum environment. After the sensor chip was aged at 300 °C for 20 h in the nitrogen environment, the varistors were measured at 25 °C–600 °C to confirm that the mesa varistors had reliable electrical connection performance at less than ≤600 °C. Finally, the encapsulated sensor chip was tested at room temperature for pressure and depressure. The test results indicated that the output sensitivity of the sensor reached ~1.09 mV/V/bar. This study provides a technical foundation for the application of SiC pressure sensors in a high-temperature environment. The sensor’s flip-chip packaging method and the pressure/decompression test at 25 °C~600 °C will be explored to promote further the development of SiC piezoresistive pressure sensors suitable for harsh environments.

## Figures and Tables

**Table 1:** Reactive ion etching machine (RIE) etching parameters of SiC.

Etching Parameters	Value
Equipment type	RIE-10NR
Etching gas	Mixture of SF_6_ and O_2_
Gas flow	SF_6_ 40 sccm, O_2_ 10 sccm
Etching power	200 W
Chamber pressure	4 Pa

**Table 2:** ICP etching parameters of SiC.

Etching Parameters	Value
Equipment type	ICP GDE C200
Etching gas	Mixture of SF_6_/O_2_/Ar_2_
Gas flow	SF_6_ 180 sccm, O_2_ 40 sccm, Ar_2_20 sccm
Etching power	RF 800W, ICP 2500W
Chamber pressure	5 mTorr

**Table 3:** Temperature coefficient of resistance (TCR) of p-type SiC.

**Temperature/(°C)**	100	200	300	400	500	600
**TCR (%/°C)**	−0.35	−0.24	−0.18	−0.14	−0.11	−0.08
